# Editorial: Unlocking brain-behavior dynamics: next-generation approaches and methods

**DOI:** 10.3389/fpsyg.2026.1725246

**Published:** 2026-02-17

**Authors:** Simone Di Plinio, Georg Northoff, Sjoerd Ebisch

**Affiliations:** 1 Department of Neuroscience Imaging and Clinical Sciences, “G. d'Annunzio” University of Chieti-Pescara, Chieti, Italy; 2 Mind, Brain Imaging and Neuroethics Research Unit, Institute of Mental Health Research, Royal Ottawa Mental Health Centre, Ottawa, ON, Canada; 3 Institute for Advanced Biomedical Technologies (ITAB), “G. d'Annunzio” University of Chieti-Pescara, Chieti, Italy

**Keywords:** brain-behavior coupling, degeneracy, electroencephalography, individual heterogeneity, neural variability, network archetypes, spatio-temporal dynamics, functional magnetic resonance imaging

We are living through a moment of renewed ambition in cognitive and affective neuroscience: a shift from siloed variables and univariate associations toward designs and analytics that respect the complexity, degeneracy, and multiscale dynamics of brain–behavior coupling. This Research Topic asked a simple but demanding question: how do we move beyond “one region, one behavior” and, instead, embrace temporal variability, interpretable machine learning, trial-level inference, electrophysiological microdynamics, and network archetypes to decode how brains give rise to diverse and sometimes convergent phenotypes? The five contributions assembled here exemplify that transition and chart practical routes for future work.

The Research Topic advances a multiscale, mechanism-aware view of brain–behavior coupling, one that treats time as signal, heterogeneity as information, and prediction as a bridge to intervention. At the temporal level, converging evidence suggests that spontaneous mentation and goal-directed control are jointly organized across scales. The contribution by Sorella et al., *Resting-State BOLD Temporal Variability of the Default Mode Network Predicts Spontaneous Mind Wandering, which is Negatively Associated with Mindfulness Skills*, reports that variability within the default mode network forecasts spontaneous mind-wandering, which in turn relates inversely to the “acting with awareness” facet of mindfulness, pointing to a specific attentional skill rather than a monolithic trait. Gupta et al.'s study, *Complementing this macroscopic lens, EEG microstates dynamics of happiness and sadness during music listening*, shows that music reshapes microstate configurations: happy music upregulates attention-related microstates (class D) and downregulates mind-wandering-related (class C), while sad music jointly elevates multiple microstates (B, C, D) and prolongs mean microstate duration, consistent with enhanced temporal stability. Read together, these findings sketch a common temporal canvas: fluctuations in large-scale resting networks and sub-second electrophysiological microstates appear to index how the brain negotiates the boundary between internally generated thought and task engagement.

Methodologically, the Topic emphasizes designs that recover small within-trial signals without inflating sample sizes. The study *Harnessing slow event-related fMRI to investigate trial-level brain–behavior relationships during object identification* (Gotts et al.) demonstrates that carefully crafted slow event-related paradigms, paired with multi-echo acquisition and ME-ICA denoising, can transform weak trial-level BOLD–RT associations into robust, replicable group-level effects with conventional sample sizes, provided each participant contributes sufficient trials. The takeaway is pragmatic and broadly applicable: optimize acquisition (multi-echo), denoise aggressively yet transparently (ME-ICA), and privilege within-participant richness to boost sensitivity while maintaining rigorous inference.

The Topic also pushes toward interpretable prediction and theory-aware mapping of heterogeneity. In a safety-critical domain, the contribution *Predicting honest behavior based on Eysenck personality traits and gender: an explainable machine learning study using SHAP analysis* (Meng et al.) leverages gradient-boosted trees with SHAP values to reveal nonlinear structure and thus highlighting neuroticism as the strongest predictor of dishonesty, threshold effects for psychoticism and extraversion, and systematic gender differences while keeping the model auditable for selection and training contexts. In parallel, the study *The degenerate coding of psychometric profiles through functional connectivity archetypes* (Di Plinio et al.) operationalizes degeneracy, that is, the many-to-many possible mapping between neural configurations and behavior, via self-organizing maps that uncover archetypal resting-state connectivity profiles whose interactions align with cognitive, physical, and socioemotional outcomes. These approaches argue that individual differences are not noise to be averaged out but structure to be modeled through local thresholds, to grasp mixture-like archetypes, and to highlight many-to-one or many-to-many solutions.

Contributions in this Topic converge on a set of emergent principles redefining how brain–behavior relationships are conceived.

- **From structure to dynamics**. Neural activity is no longer understood as a static architecture but as a temporally evolving system whose fluctuations encode meaning. Variability is itself an informative signal, capturing the adaptive transitions between internal and external modes of cognition.
- **From average effects to individualized trajectories**. Rather than chasing ever-larger samples to stabilize small effects, we shift toward within-participant nuance to allow the modeling of trial-level and state-level variability. This reframing treats individuals not as noise around a mean but as a dynamic system contributing its own lawful regularities.
- **From prediction to understanding**. Integrating of explainable machine learning and archetypal modeling demonstrates that high predictive accuracy can coexist with psychological interpretability. Models now aspire not only to forecast behavior but to illuminate the neural routes underneath.
- **From uniformity to degeneracy**. Behavioral equivalence can emerge from multiple neural configurations. Recognizing this degeneracy transforms variability from a methodological nuisance into a biological feature encoding resilience, plasticity, and diversity.
- **From observation to modulation**. By linking dynamic neural signatures to trainable skills, these studies open the door to targeted interventions that refine rather than suppress variability, suggesting that the next generation of neurobehavioral research will be not only descriptive but transformative.

Together, these principles illustrate three converging shifts that define next-generation neuroscience: time is mechanistic, revealing how neural variability orchestrates transitions between spontaneous thought and control; design beats despair, demonstrating that methodological precision can recover meaningful signal without unmanageable scale; and interpretability enables action, ensuring that complex models remain transparent, ethically accountable, and useful for prediction and intervention alike.

In sum, this Research Topic reframes brain–behavior science from static localization to process-aware, heterogeneity-savvy, and intervention-ready inference. By integrating temporal dynamics (fMRI variability, EEG microstates), optimized experimental design (multi-echo, ME-ICA, trial density), and interpretable modeling (SHAP, archetypes, degeneracy), these studies outline a toolkit for decoding how different brains achieve similar behaviors and how we might steer those dynamics ethically and effectively.

When considered in a more general and abstract way, these results demonstrate that cognitive functions are strongly shaped by the spatial-topographic and temporal-dynamic features of our brain's neural activity, reflecting the importance of topics such as Spatiotemporal Neuroscience and degeneracy in brain-behavioral coding.

